#  Langerhans Cell Migration in Murine Cutaneous Leishmaniasis: Regulation by Tumor Necrosis Factor α Interleukin-1β, and Macrophage Inflammatory Protein-1α

**DOI:** 10.1155/1998/21095

**Published:** 1998

**Authors:** Jörg Arnoldi, Heidrun Moll

**Affiliations:** 1 Research Center for Infectious Diseases University of Würzburg Würzburg D-97070 Germany; 2 Research Center for Infectious Diseases University of Würzburg Röntgenring 11 Würzburg D-97070 Germany

**Keywords:** Langerhans cells, migration, leishmaniasis, cytokines, chemokines

## Abstract

After intradermal infection of mice with the obligatory intracellular parasite *Leishmania major*, Langerhans cells (LC) are intimately involved in the induction of the primary T-cell immune response. LC can phagocytose *Leishmania* and transport ingested parasites from the infected skin to the regional lymph nodes. Since TNFα and IL-1β have been shown to induce LC migration after epicutaneous exposure to skin-sensitizing chemicals, we investigated the involvement of both cytokines in the migration of *Leishmania*-infected LC. In addition, the relevance of two chemokines of the β subfamily, macrophage inflammatory protein 1α(MIP-1α) and macrophage chemoattractant protein 1 (MCP-1), was analyzed.*In vivo* depletion of TNFα significantly reduced the amount of infected LC and the parasite load in the draining lymph nodes. Administration of recombinant TNFα caused the reverse effect. In contrast, the depletion of IL1β enhanced the parasite-induced LC migration, whereas treatment with recombinant IL-1β, as well as recombinant MIP- c, reduced the rate of infected LC in the lymph nodes. MCP- did not influence LC migration. Our data demonstrate that TNFα and IL-1β are regulating the LCmediated transport of *Leishmania* and also provide evidence for the involvement of macrophage attractant chemokines in this process.


*Developmental Immunology*, 1998, Vol. 6, pp. 3–11
Reprints available directly from the publisher
Photocopying permitted by license only

© 1998 OPA (Overseas Publishers Association)
N.V. Published by license under the
Harwood Academic Publishers imprint,
part of the Gordon and Breach Publishing Group
Printed in Malaysia

### Langerhans Cell Migration in Murine Cutaneous Leishmaniasis: Regulation by Tumor Necrosis Factor $\alpha$, Interleukin-1$\beta$, and Macrophage Inflammatory Protein-1$\alpha$

JÖRG ARNOLDI and HEIDRUN MOLL*

*Research Center for Infectious Diseases, University of Würzburg, D-97070 Würzburg, Germany*

*(Received 16 August 1996; Accepted 14 August 1997)*

After intradermal infection of mice with the obligatory intracellular parasite *Leishmania major*, Langerhans cells (LC) are intimately involved in the induction of the primary T-cell immune response. LC can phagocytose *Leishmania* and transport ingested parasites from the infected skin to the regional lymph nodes. Since TNF$\alpha$ and IL-1$\beta$ have been shown to induce LC migration after epicutaneous exposure to skin-sensitizing chemicals, we investigated the involvement of both cytokines in the migration of *Leishmania*-infected LC. In addition, the relevance of two chemokines of the $\beta$ subfamily, macrophage inflammatory protein 1$\alpha$ (MIP-1$\alpha$) and macrophage chemoattractant protein 1 (MCP-1), was analyzed. *In vivo* depletion of TNF$\alpha$ significantly reduced the amount of infected LC and the parasite load in the draining lymph nodes. Administration of recombinant TNF$\alpha$ caused the reverse effect. In contrast, the depletion of IL-1$\beta$ enhanced the parasite-induced LC migration, whereas treatment with recombinant IL-1$\beta$, as well as recombinant MIP-1$\alpha$, reduced the rate of infected LC in the lymph nodes. MCP-1 did not influence LC migration. Our data demonstrate that TNF$\alpha$ and IL-1$\beta$ are regulating the LC-mediated transport of *Leishmania* and also provide evidence for the involvement of macrophage attractant chemokines in this process.

*Keywords:* Langerhans cells, migration, leishmaniasis, cytokines, chemokines

### INTRODUCTION

Epidermal Langerhans cells (LC), as members of the dendritic-cell lineage, are part of a system of potent antigen-presenting cells in skin and lymph nodes (Steinman, 1991). Dendritic cells constitutively express MHC class II molecules and have the unique ability to induce primary T-cell responses. Upon stimulation with skin-irritating chemicals or after skin transplantation, LC leave the skin and migrate via the

*Corresponding author. Present address: Research Center for Infectious Diseases, University of Würzburg, Röntgenring 11, D-97070 Würzburg, Germany lymphatics into the lymph nodes, draining the site of epicutaneous exposure (Macatonia et al., 1987; Larsen et al., 1990). During this migration, LC are subject to a functional and phenotypic differentiation that is thought to be initiated by epidermal cytokines. They develop into highly efficient antigen-presenting cells with the potential to induce the primary stimulation of specific T cells.

Recent studies revealed that the induction of an antigen-specific T-cell immune response after cutaneous infection with the obligatory intracellular parasite *Leishmania major* is mediated by epidermal LC (Moll et al., 1993, 1995). LC can phagocytose *L. major* and transport them from the skin to the local lymph nodes for presentation of parasite antigen to resting T cells. LC produce cytokines such as interleukin-1$\beta$ (IL-1$\beta$) and the chemokine macrophage inflammatory protein 1$\alpha$ (MIP-1$\alpha$) (Heufler et al., 1992; Schreiber et al., 1992). Furthermore, keratinocytes in the skin epidermis release tumor necrosis factor $\alpha$ (TNF$\alpha$) and granulocyte/macrophage-colony stimulating factor (GM-CSF) that are critical for the viability and differentiation of LC (Koch et al., 1990). However, the factors regulating LC migration are not completely understood. In this study, we analyzed the role of TNF$\alpha$ and IL-1$\beta$ in *Leishmania*-induced LC migration. Furthermore, the influence of the chemokines MIP-1$\alpha$ and macrophage chemoattractant protein 1 (MCP-1) was investigated. Both chemokines are involved in skin-associated inflammatory processes and were recently shown to be important markers for the classification of different clinical forms of human cutaneous leishmaniasis (Ritter et al., 1996). Recombinant cytokines or neutralizing antibodies were administered *in vivo*, prior to or simultaneously with infection with the parasite. The amount of infected LC migrating to the lymph nodes was estimated by double-staining techniques. In addition, the frequency of parasite-infected cells in the regional lymph nodes was determined in a limiting dilution assay. To distinguish the effects of locally produced mediators from those of exogenous sources on LC migration, we additionally used a skin explant culture system.

### RESULTS

### Detection of *L. major*-Containing Dendritic Cells

In the first experiments, we defined the time point that is suitable for detection of parasite-bearing LC arriving in lymph nodes draining the site of infection. As early as 2 days after intradermal infection of mice with *L. major* promastigotes, significant numbers of parasite-containing cells could be identified in lymph node sections using a double-staining technique (Figure 1). This time point was used in all experiments to evaluate the effects of TNF$\alpha$, IL-1$\beta$, MIP-1$\alpha$, and MCP-1 on LC migration. It is of particular importance that at this early time point, *Leishmania* parasites only can be found in lymph node dendritic cells and are not associated with macrophages (Moll et al., 1993).

### TNF$\alpha$ Enhances the *Leishmania*-Induced LC Migration

To determine whether TNF$\alpha$ has an effect on the migration rate of infected LC, mice were treated with recombinant TNF$\alpha$ and, at the same time, were infected intradermally with *L. major* parasites. Two days after treatment, the lymph nodes draining the site of infection were removed and immunohistochemical double staining was performed to enumerate infected dendritic cells. Figure 2 illustrates that the administration of recombinant TNF$\alpha$ enhanced the number of parasite-bearing dendritic cells in the lymph nodes nearly twofold as compared to control mice that received normal serum. In order to confirm the involvement of TNF$\alpha$ in the induction of LC migration, mice were treated with neutralizing anti-TNF$\alpha$ antibodies, 2 hr before infection with the parasite. The depleting antibodies were applied intraperitoneally and not locally to avoid antibody-induced changes in the content of LC at the site of exposure to the parasite (unpublished observation). As shown in Figure 2, the *in vivo* depletion of TNF$\alpha$ resulted in a pronounced reduction of infected LC in the lymph nodes. It should be noted that LC migration could not be totally inhibited by *in vivo* depletion of TNF$\alpha$.

significantly change the frequency of infected lymph node cells. Thus, the cytokine-induced changes in LC migration correlate with those in the parasite load of the draining lymph nodes.

### Skin Explant Cultures Show that TNF$\alpha$ and IL-1$\beta$ Act as Local Mediators in the Skin

Next, we wanted to investigate whether the influence of TNF$\alpha$ and IL-1$\beta$ on LC migration is dependent on the infection with *Leishmania* parasites. Furthermore, we wanted to analyze the effects of cytokine treatment on the local environment in the skin. To exclude the involvement of exogenous mediators, released by infiltrating cells, we harvested the skin areas immediately after administration of recombinant TNF$\alpha$ or IL-1$\beta$ alone or in combination with *L. major* promastigotes. The skin explants were placed onto medium in 3-ml wells and, after 4 days of culture, the emigrated cells were immunostained for dendritic cell markers. As demonstrated in Table 2, the administration of IL-1$\beta$, TNF$\alpha$, parasites or phoshate-buffered saline (PBS) alone induce an enhanced LC emigration, as compared to untreated controls. Interestingly, the amount of emigrated dendritic cells was significantly lower than the PBS-treated group after combined treatment with *L. major* parasites and recombinant IL-1$\beta$. On the other hand, only the combined administration of TNF$\alpha$ with *Leishmania* parasites significantly enhanced the rate of emigrated LC. Thus, these data confirm the results obtained from the analysis of lymph nodes and, in addition, show that TNF$\alpha$ acts locally to enhance the LC-mediated export of *L. major* from the skin, whereas IL-1$\beta$ reduces this process.

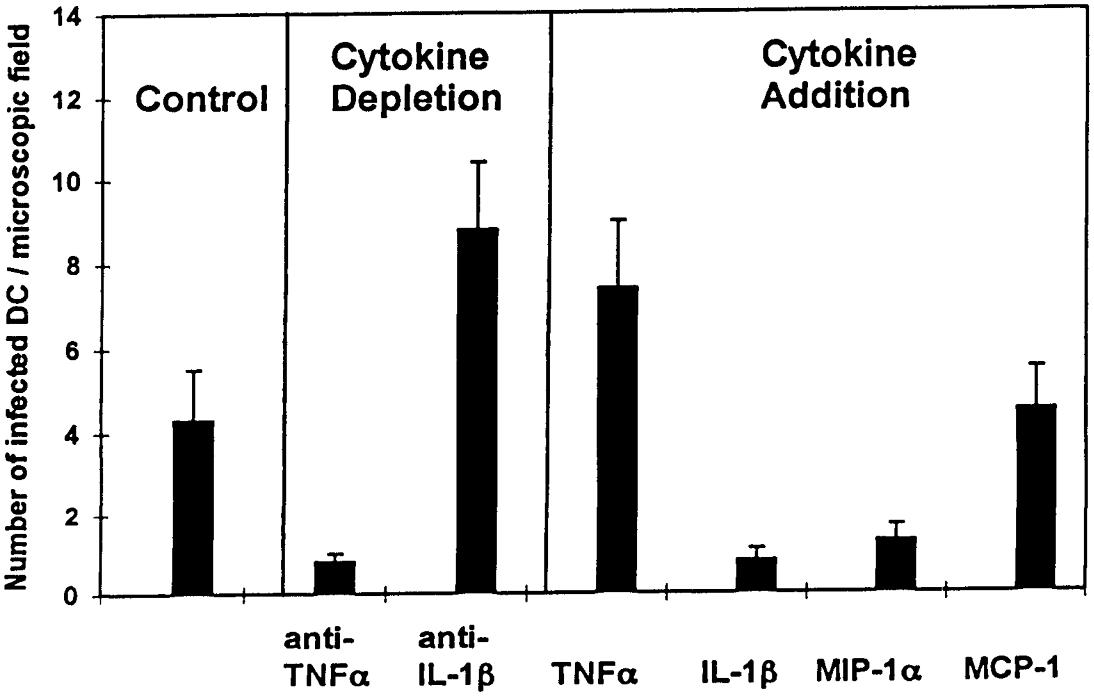

FIGURE 2 The migration of *L. major*-infected LC is modulated by cytokines and chemokines. The microscopical quantification of infected LC in lymph node sections after administration of control serum, neutralizing antibodies, or recombinant cytokines/chemokines and intradermal infection with $1 \times 10^6$ *L. major* promastigotes. Forty-eight hours after infection, serial sections were subjected to double-staining immunohistology for detection of NLDC-145$^+$ cells expressing *L. major* antigen. The data represent means ± SD of 10 random fields at ×400 magnification. Two additional experiments gave similar results.

TABLE I Frequency of Lymph Node Cells Infected with *Leishmania* Parasites

| Treatment of mice | Frequency ($\times 10^6$) | 95% Confidence limits (reciprocal values) |
|---|---|---|
| Control serum | 1/8.5 | 6.4-9.0 |
| Anti-TNF$\alpha$ | 1/33.9 | 20.5-68.9 |
| rTNF$\alpha$ | 1/4.5 | 3.5-6.2 |
| Anti-IL-1$\beta$ | 1/2.2 | 1.6-3.1 |
| rIL-1$\beta$ | 1/24.2 | 15.6-53.5 |
| rMIP-1$\alpha$ | 1/12.7 | 10.2-22.1 |
| rMCP-1 | 1/6.8 | 5.3-9.9 |

*Note*: Limiting dilution analysis of total lymph node cells after administration of neutralizing antibodies, recombinant cytokines/chemokines, or control serum and intradermal infection with $1 \times 10^6$ *L. major* promastigotes. Forty-eight hours after infection, single-cell suspensions were prepared and serial dilutions were incubated in blood agar cultures. Seven days later, the proportion of *Leishmania*-negative cultures was determined. The experiments were repeated three times with similar results.

TABLE II Emigration of LC from Skin Explant Cultures

| *In vivo* treatment | Number of NLDC-145$^+$ cells/100 cells |
|---|---|
| None | 0.5 ± 0.08 |
| PBS | 1.6 ± 0.5 |
| rIL-1$\beta$ | 1.8 ± 0.3 |
| *L. major* + rIL-1$\beta$ | 0.8 ± 0.1 |
| rTNF$\alpha$ | 2.2 ± 0.4 |
| *L. major* + rTNF$\alpha$ | 2.6 ± 0.4 |

*Note*: Microscopical evaluation of epidermal dendritic cells that have emigrated from skin explants. Prior to culture in Click's RPMI medium, the skin was intradermally infected with $1 \times 10^6$ *L. major* parasites. Simultaneously, recombinant cytokines were administered. Four days after skin explantation, emigrated dendritic cells were harvested from the bottom of the wells and immunocytochemically detected by the APAAP technique. The data represent means ± SD of 10 random fields at ×400 magnification and two sets of experiments.

### DISCUSSION

The rapid emigration of LC from the epidermis and their consecutive accumulation in the paracortical areas of the draining lymph nodes after sensitization with alloantigens or contact allergens has been demonstrated previously in different systems (Macatonia et al., 1987; van Wilsem et al.; 1994, Kimber and Cumberbatch, 1995). Recent studies have extended these observations by showing that not only allergens, but also the skin-associated protozoan parasite *L. major* can induce this migration (Moll et al., 1993). Cytokines, in particular TNF$\alpha$, have been suggested to be involved in this phenomenon. Following topical exposure to skin-sensitizing chemicals, the administration of TNF$\alpha$, a potent immunomodulatory molecule, causes a reduction in the density of LC in the skin and an increase in the number of antigen-bearing LC in the draining lymph nodes in a concentration- and time-dependent manner (Cumberbatch and Kimber, 1992). The administration of neutralizing anti-TNF$\alpha$ antibodies, on the other hand, inhibits dendritic cell accumulation in lymph nodes (Cumberbatch and Kimber, 1995). Furthermore, TNF$\alpha$ was shown to directly influence LC function by maintaining their viability in culture (Koch et al., 1990). Our results presented here, using the skin-associated parasite *L. major*, extend these data to an infectious disease model and provide strong evidence that TNF$\alpha$ is an important signal inducing the migration of antigen-bearing LC in cutaneous leishmaniasis. The *in vivo* depletion of TNF$\alpha$ caused a pronounced reduction in the number of infected dendritic cells and the parasite load in the draining lymph nodes. As demonstrated *in vivo* and in skin explant cultures, the administration of recombinant TNF$\alpha$ caused the reverse effect. These data show that TNF$\alpha$ is a locally induced mediator of LC migration not only after contact sensitization, but also after intradermal infection with parasites. A recent model to explain LC migration after antigenic stimulation is based on the interaction of LC with keratinocytes, which are known to be the major source of TNF$\alpha$ in the skin (Schreiber et al., 1992). Since LC have been suggested to express the TNF receptor type II (p75), they may respond to TNF released from keratinocytes (Wang et al., 1996). This may result in the activation of protein kinase C, which transduces the signal for LC migration from the epidermis (Halliday and Lucas, 1993).

Epidermal LC have been identified as the major source of IL-1$\beta$ production in the epidermis (Heufler et al., 1992; Schreiber et al., 1992). Following epicutaneous skin sensitization, IL-1$\beta$ secretion can be induced within minutes. Paracrine and autocrine mechanisms cause the upregulation of MHC class II expression on LC and enhancement of their immunostimulatory potential for resting T cells (Heufler et al., 1988, Nylander-Lundqvist and Bäck, 1990). This is consistent with the finding that the *in vivo* depletion of IL-1$\beta$ by monoclonal antibodies completely prevents sensitization to allergens (Enk et al., 1993). Furthermore, there is evidence that IL-1$\beta$ can induce LC migration (Kämpgen et al., 1995). These findings were further substantiated by the use of IL-1$\beta$-deficient mice, which showed defective contact hypersensitivity responses to topically applied skin sensitizers (Shornick et al., 1996). In the present study, we analyzed the role of IL-1$\beta$ in *Leishmania*-induced LC migration by the use of recombinant IL-1$\beta$ or neutralizing anti-IL-1$\beta$ antibodies. Administration of the recombinant cytokine was expected to enhance the parasite-induced LC migration. Interestingly, however, the injection of IL-1$\beta$ reduced the migration rate of *L. major*-infected LC *in vivo*. The depletion of the cytokine caused the reverse effect. As demonstrated in skin explant cultures, this phenomenon was strongly dependent on the presence of parasites, because the application of IL-1$\beta$ alone resulted in an enhancement of LC emigration and, thus, had an effect that was similar to the nonspecific response after PBS injection. The observed reduction in the number of parasite-containing LC leaving the skin and reaching the lymph node after treatment with IL-1$\beta$ can most likely be explained by a decrease in the phagocytic activity of LC. The presence of recombinant IL-1$\beta$ significantly reduced the proportion of *L. major*-infected LC *in vitro* in a dose-dependent manner (data not shown). Blocking this inhibitory effect of IL-1$\beta$ *in vivo*, by administration of anti-IL-1$\beta$ antibodies, may result in an enhanced capacity to phagocytose parasites, and in consequence would increase the percentage of infected LC migrating to the lymph nodes. Therefore, our findings suggest that the detrimental role of IL-1$\beta$ in *L. major* infection is not only caused by the selective activation of T helper 2 over T helper 1 cells (Chakkalath and Titus, 1994), but is also due to a decreased rate of parasite phagocytosis and transport by LC in the very early phase of infection, which is critical for the primary stimulation of specific T cells.

The chemokine MIP-1$\alpha$, primarily secreted by stimulated macrophages, additionally is expressed by epidermal LC (Matsue et al., 1992). MIP-1$\alpha$ causes local inflammatory reactions and can regulate epidermal homeostasis by inhibiting keratinocyte colony formation. Thus, LC, as the major source of MIP-1$\alpha$ in the epidermis, can downregulate keratinocyte proliferation (Heufler et al., 1992). Keratinocytes, the overwhelming majority of epidermal cells, have been shown to be important inducers of LC maturation and differentiation (reviewed in Kämpgen et al., 1995). This implies that inhibition of keratinocyte function results in a reduced ability to stimulate LC and, in consequence, may diminish the antigen-induced differentiation and migration of LC. This hypothesis is in line with our data demonstrating that intradermal administration of MIP-1$\alpha$, in combination with *L. major* parasites, decreased the number of antigen-bearing dendritic cells and the parasite load in the draining lymph nodes.

In conclusion, our observations extend the important roles of TNF$\alpha$, IL-1$\beta$, and MIP-1$\alpha$ in the regulation of LC migration to an infectious disease model. They also show that IL-1$\beta$ has a differential role in this process, because it supports LC migration in contact sensitization with haptens, whereas it inhibits the transport of the intracellular parasite *L. major* by reducing the phagocytic activity of LC.

### MATERIAL AND METHODS

### Mice

Female mice of the inbred strain BALB/c were 8 to 10 weeks of age at the onset of experiments. All mice were purchased from Charles River Breeding Laboratories (Sulzfeld, Germany) and, during experimentation, were maintained under specific pathogen-free (SPF) conditions in an isolation facility.

### Parasites and Infection of Mice

The origin, culture, and propagation of the *L. major* isolate (MHOM/IL/81/FE/BNI) have been described elsewhere (Solbach et al., 1986). Promastigotes were grown *in vitro* in blood agar cultures. Stationary-phase promastigotes were washed in PBS and, for intradermal infection of mice, $1 \times 10^6$ organisms were injected in a volume of 10 $\mu$l on the dorsum, using a 100-$\mu$l Hamilton syringe mounted with a 30-gauge Yale needle.

### Antibodies and Cytokines

Polyclonal rabbit anti-mouse TNF$\alpha$ antiserum, recombinant murine TNF$\alpha$, and IL-1$\beta$ were purchased from Genzyme (Cambridge, MA), and goat anti-murine IL-1$\beta$ was a gift from R&D systems (Wiesbaden, Germany). Recombinant MIP-1$\alpha$ and MCP-1 were products from R&D systems. Rat monoclonal antibodies (mAb) against nonlymphoid dendritic cells, from hybridoma NLDC-145, as well as the isotype control antibodies, were from Dianova (Hamburg, Germany). Polyclonal antibodies to *L. major* were raised in rabbits by subcutaneous and intramuscular injections of promastigotes in Complete Freund's adjuvans (CFA) followed by several boosters of promastigotes in Incomplete Freund's adjuvans (IFA) and collection of the serum. Goat anti-rat immunoglobulin antiserum was obtained from Dianova, and alkaline phoshatase anti-alkaline phosphatase (APAAP) complexes were purchased from Dako (Hamburg, Germany). An immunogold-conjugated goat anti-rabbit immunglobulin antibody (Amersham, Braunschweig, Germany) served as second-stage reagent for silver-enhanced staining.

### Animal Treatment

All animal manipulations, other than antibody treatment, were performed under metofane anesthesia (Janssen, Neuss, Germany). Antibodies against TNF$\alpha$ and IL-1$\beta$ were injected intraperitoneally (1 mg/mouse) 2 hr before infection with *L. major* parasites. The recombinant cytokines and chemokines were injected intradermally (50 ng/mouse) simultaneously with the parasites. Control mice received normal serum at equivalent concentrations. All preparations were diluted with sterile PBS and administered in a total volume of 10 $\mu$l.

### Skin Explant Culture

Immediately after intradermal injections of *L. major* promastigotes and/or recombinant cytokines onto the shaved dorsum of BALB/c mice, skin explants of 10 $mm^2$ were transferred to 12-well plates and cultured in Clicks RPMI containing 10% FCS. After 4 days, the emigrated cells were harvested from the bottom of the wells and cytospins were prepared for subsequent immunostaining.

### Immunostaining

For immunohistological identification of *L. major*-containing cells, lymph nodes were snap-frozen in OCT compound (Miles, Naperville, IL). Cryostat sections (4-6 $\mu$m) as well as cytospins were fixed in 4% paraformaldehyde for 20 min, followed by extensive washings in PBS. Nonspecific binding sites were blocked by incubation in Blotto (PBS containing 10% skim milk powder and 0.1% bovine serum albumin [BSA], fraction V) supplemented with 10% FCS for 30 min at room temperature. Mixed labeling of tissue sections was performed by incubation with the primary antibodies diluted in Blotto for 1 hr followed by treatment with goat anti-rat immunoglobulin antiserum diluted in Blotto (30 min) and the APAAP complex diluted in PBS (30 min). After incubation with immunogold-labeled immunoglobulin antiserum diluted in Blotto for 30 min, the APAAP staining was developed with New Fuchsin (Cordell et al., 1984). After postfixation in 2% glutaraldehyde and extensive washings in aqua bidest, immunogold signals were detected by silver enhancement according to the manufacturer's instructions (Amersham). Finally, slides were counterstained with hematoxylin and mounted in Kaisers glycerin gelatine (Merck, Darmstadt, Germany).

All immunostainings were controlled by (1) the use of secondary reagents alone, to confirm their species specificity; (2) the development of alkaline phosphatase alone, to exclude staining due to endogeneous enzyme activity; and (3) the use of rat and rabbit isotype control antibodies. All control stainings yielded negative results.

### Limiting Dilution Assay

Groups of three mice were used to determine the parasite load in the draining lymph nodes, 2 days after intradermal infection with *L. major*. Single-cell suspensions were prepared and lymph node cells were resuspended in Clicks RPMI containing 10% FCS. Serial dilutions ranging from $1 \times 10^8$ to $1 \times 10^3$ cells/well (20 replicate cultures per dilution) were incubated in blood agar microcultures to support the growth of parasites. After 7 days of incubation at 28°C, the cultures were scored for the presence of parasites using an inverted microscope. Minimal estimates of the frequencies of *L. major*-infected cells and the 95% confidence intervals were calculated according to the minimum $\chi^2$ method from the Poisson distribution relationship between the number of lymph node cells and the logarithm of the fraction of *Leishmania*-negative cultures by using a computer program (Taswell, 1987).

### *Acknowledgments*

The authors are grateful to R&D systems, Germany, for providing anti-IL-1$\beta$ antibodies and to Dr. Jan Kimber, ZENECA Central Toxicology Laboratory, Cheshire, UK, and thank Sandra Köster for excellent technical assistence. This work was supported by the Bundesministerium für Bildung, Wissenschaft, Forschung und Technologie (BMBF grant KI 8906/0).

### *References*

Blank C., Fuchs H., Rappersberger K., Röllinghoff M., and Moll H. (1993). Parasitism of epidermal Langerhans cells in experimental cutaneous leishmaniasis with *Leishmania major*. J. Infect. Dis. **167**: 418-425.

Chakkalath H.R., and Titus R.G. (1994). *Leishmania major*-parasitized macrophages augment Th2-type T cell activation. J. Immunol. **153**: 4378-4387.

Cordell J.L., Fallini B., Erber W.N., Ghosh A.K., Abdulaziz Z., MacDonald S., Pulford K.A.F., Stein H., and Mason D.Y. (1984). Immunoenzymatic labeling of monoclonal antibodies using immune complexes of alkaline phosphatase and monoclonal anti-alkaline phosphatase (APAAP complexes). J. Histochem. Cytochem. **32**: 219-229.

Cumberbatch M., and Kimber I. (1992). Dermal tumour necrosis factor-$\alpha$ induces dendritic cell migration to draining lymph nodes, and possibly provides one stimulus for Langerhans' cell migration. Immunology **75**: 257-263.

Cumberbatch M., and Kimber I. (1995). Tumour necrosis factor-$\alpha$ is required for accumulation of dendritic cells in draining lymph nodes and for optimal contact sensitization. Immunology **84**: 31-35.

Enk A.H., Angeloni V.L., Udey M.C., and Katz S.I. (1993). An essential role for Langerhans cell-derived IL-1$\beta$ in the initiation of primary immune responses in skin. J. Immunol. **150**: 3698-3704.

Halliday G.M., and Lucas A.D. (1993). Protein kinase C transduces the signal for Langerhans cell migration from the epidermis. Immunology **79**: 621-626.

Heufler C., Koch F., and Schuler G. (1988). Granulocyte-macrophage colony-stimulating factor and interleukin-1 mediate the the maturation of murine epidermal Langerhans cells into potent immunostimmulatory dendritic cells. J. Exp. Med. **167**: 700-705.

Heufler C., Topar G., Koch F., Trockenbacher B., Kämpgen E., Romani N., and Schuler G. (1992). Cytokine gene expression in murine epidermal cell suspensions: Interleukin 1$\beta$ and macrophage inflammatory protein 1$\alpha$ are selectively expressed in Langerhans cells but are differentially regulated in culture. J. Exp. Med. **176**: 1221-1226.

Kämpgen E., Romani N., Koch F., Eggert A., and Schuler G. (1995). Cytokine receptors on epidermal Langerhans cells. In The Immune Functions of Epidermal Langerhans Cells, Moll H., Ed. (City: R.G. Landes), pp. 37-72.

Kimber I., and Cumberbatch M. (1995). Langerhans cell migration: Initiation and regulation. In The Immune Functions of Epidermal Langerhans Cells, Moll H., Ed. (City: R.G. Landes), pp. 103-117.

Koch F., Heufler C., Kämpgen E., Schneeweiss D., Bock G., and Schuler G. (1990). Tumor necrosis factor $\alpha$ maintains the viability of murine epidermal Langerhans cells in culture, but in contrast to granulocyte/macrophage colony-stimulating factor, without inducing their functional maturation. J. Exp. Med. **171**: 159-172.

Larsen C.P., Steinman R.M., Witmer-Pack M., Hankins D.F., Morris P.J., and Austyn J.M. (1990). Migration and maturation of Langerhans cells in skin transplants and explants. J. Exp. Med. **172**: 1483-1493.

Macatonia S.E., Knight S.C., Edwards A.J., Griffiths S., and Fryer P. (1987). Localization of antigen on lymph node dendritic cells after exposure to the contact sensitizer fluorescein isothiocyanate. Functional and morphological studies. J. Exp. Med. **166**: 1654-1667.

Matsue H., Cruz P.D., Bergstresser P.R., and Takashima A. (1992). Langerhans cells are the major source of mRNA for IL-1$\beta$ and MIP-1$\alpha$ among unstimulated mouse epidermal cells. J. Invest. Dermatol. **99**: 537-541.

Moll H., Fuchs H., Blank C., and Röllinghoff M. (1993). Langerhans cells transport *Leishmania major* from the infected skin to the draining lymph node for presentation to antigen-specific T cells. Eur. J. Immunol. **23**: 1595-1601.

Moll H., Flohé S., and Röllinghoff M. (1995). Dendritic cells in *Leishmania major*-immune mice harbor persistent parasites and mediate an antigen-specific T cell immune response. Eur. J. Immunol. **25**: 693-699.

Nylander-Lundqvist E., and Bäck O. (1990). Interleukin-1 decreases the number of Ia$^+$ epidermal dendritic cells but increases their expression of Ia antigen. Acta Derm. Venereol. (Stockholm). **70**: 391-394.

Ritter U., Moll H., Laskay T., Bröcker E-B., Velazco O., Becker I., and Gillitzer R. (1996). Differential expression of chemokines in patients with localized and diffuse cutaneous American leishmaniasis. J. Infect. Dis. **173**: 699-709.

Schreiber S., Kilgus O., Payer E., Kutil R., Elbe A., Mueller C., and Stingl G. (1992). Cytokine pattern of Langerhans cells isolated from murine epidermal cell cultures. J. Immunol. **149**: 3525-3534.

Shornick L.P., De Togni P., Mariathasan S., Goellner J., Strauss-Schoenberger J., Karr R.W., Ferguson T.A., and Chaplin D.D. (1996). Mice deficient in IL-1$\beta$ manifest impaired contact hypersensitivity to Trinitrochlorobenzene. J. Exp. Med. **183**: 1427-1436.

Solbach W., Forberg K., and Röllinghoff M. (1986). Effect of T-lymphocyte suppression on the parasite burden in *Leishmania major*-infected, genetically susceptible BALB/c mice. Infect. Immunol. **54**: 909-912.

Steinman R.M. (1991). The dendritic cell system and its role in immunogenicity. Annu. Rev. Immunol. **9**: 271-296.

Taswell C. (1987). Limiting dilution assays for the separation, characterization and quantitation of biologically active particles and their clonal progeny. In Cell Separation: Methods and Selected Applications, Pretlow T.G. and Pretlow T.P., Eds. (Orlando, FL: Academic Press), pp. 109-145.

Wang B., Kondo S., Shivji G.M., Fujisawa H., Mak T.W., and Sauder D.N. (1996). Tumour necrosis factor receptor II (p75) signalling is required for the migration of Langerhans' cells. Immunology **88**: 284-288.

Van Wilsem E.J.G., Breve J., Kleijmeer M., and Kraal G. (1994). Antigen-bearing Langerhans cells in skin draining lymph nodes: Phenotype and kinetics of migration. J. Invest. Dermatol. **103**: 217-220.

